# Case Management of COVID-19 (Secondary Version)

**DOI:** 10.31662/jmaj.2021-0036

**Published:** 2021-07-09

**Authors:** Yasuyuki Kato

**Affiliations:** 1 Department of Infectious Diseases, International University of Health and Welfare (IUHW) Narita Hospital, Chiba, Japan

**Keywords:** COVID-19, disease severity, drug therapy, clinical trials, steroids

## Abstract

Case management of COVID-19 is critically important to save lives and reduce the fear and anxiety evoked by this disease in communities. However, most healthcare systems have been overwhelmed in many parts of the world. It is also essential to screen patients to be able to identify those who are likely to suffer from severe disease, to ensure more effective use of medical resources. Disease severity can be determined based on simple vital signs; some laboratory markers are useful adjuncts.

Treatment for COVID-19 is largely supportive although a number of repurposed drugs have been evaluated for its efficacy in clinical trials. For example, dexamethasone has now been considered as a standard therapy for severe cases, while remdesivir and tocilizumab are promising agents in selected patient groups.

## I．Introduction

Coronavirus disease 2019 (COVID-19) is defined as an acute respiratory infection caused by severe acute respiratory syndrome coronavirus 2 (SARS-CoV-2) ^(1), (2)^. Its first case was reported in Wuhan, China, in December 2019 and, since then, has spread across the globe. As of March 7, 2021, about 116 million confirmed cases of COVID-19 have been recorded worldwide, including around 2.6 million deaths, as per the reports of the World Health Organization (WHO) ^(3)^. The clinical picture is broad, ranging from asymptomatic infection to upper respiratory tract infections, pneumonia, acute respiratory distress syndrome, and multiple organ failure ^(4)^. Majority of the fatal cases have been observed among older people or people who have underlying diseases ^(5)^.

Thus, case management of COVID-19 is critically important to save lives and reduce the fear and anxiety evoked by this disease in communities. However, most healthcare systems have been overwhelmed in many parts of the world ^(6)^. Treatment for COVID-19 is largely supportive although a number of repurposed drugs have been evaluated for its efficacy in clinical trials ^(7)^. Through these concerted efforts, dexamethasone has now been considered as a standard therapy for severe cases who require supplemental oxygen although many clinicians raised safety concerns for its use in the initial phase of the pandemic. In this paper, the clinical management of COVID-19 patients with mild to moderate disease is discussed focusing on Japanese clinical settings ^(8)^.

## II．Management Based on Disease Severity

Patients suspected of having COVID-19 are required to undergo diagnostic testing for confirmation of the disease (polymerase chain reaction or antigen test). After confirming a diagnosis, it is also essential to screen the patients to identify those who are likely to suffer from severe disease, to ensure more effective use of medical resources. During epidemics, it is vital to quickly and thoroughly take one’s medical history to obtain pertinent facts such as date of onset of symptoms, age and sex of the patient, and presence of any underlying disease.

Disease severity can be determined based on simple vital signs, including level of consciousness and respiratory rate as that of community-acquired pneumonia ^(7), (8)^. However, some patients with COVID-19 do not complain of dyspnea despite the presence of respiratory failure (silent hypoxia); thus, it is desirable to measure percutaneous oxygen saturation (SpO_2_) using a pulse oxymeter ^(9)^. Patients with SpO_2_ lower than 96% are suspected of having pneumonia and should thus be assessed further ^(8)^. Blood tests and imaging modalities add benefits to verify disease severity. Computed tomography (CT) is the method of choice for the early detection of pneumonia findings (ground-glass opacities) ^(10)^. Once the disease severity is identified based on SpO_2_ and the patient’s clinical condition, a treatment policy is decided on (Table 1). As regards blood test results for assessment of disease severity, high levels of urea nitrogen (>20 mg/dL), C-reactive protein (CRP > 5.0 mg/dL), lactate dehydrogenase (>500 IU/L), ferritin (>1,000 μg/L), and D-dimer (>2.0 μg/mL) often correlate with poor prognosis and thus can be used as a reference ^(11), (12)^.

**Table 1.** Management of COVID-19 Patients Based on Disease Severity.

	Definitions	Place of medical care	Points of medical care
Mild	SpO_2_ > 96 With no evidence of pneumonia	Home Medical institution (elderly, with underlying illness)	Persons should be observed from the day of onset up to 10 days. Symptoms may appear even in asymptomatic carriers. Regularly measure SpO_2 _in the elderly or in those with underlying illness to detect early progression to moderate or more severe disease. Olfactory and taste disorders are likely to persist.
Moderate I	93 < SpO_2_ < 96	Medical institution	Careful follow-up is required using respiratory rate and SpO_2_ as indicators. In patients with decreased SpO_2_, oxygen administration is started, and drug therapy (a combination of remdesivir and steroids) is recommended. Screening for thrombosis is also performed. Bacterial infections are rare, and the need for antibiotics is assessed on a case-by-case basis.
Moderate II	SpO_2_ < 93	Medical institution	
Severe	Requires artificial ventilation	Advanced medical institution designated under the coordination of prefectures	It is desirable to receive treatment at an appropriate medical institution.

COVID-19, coronavirus disease; SpO_2_, percutaneous oxygen saturation

The indication for hospitalization according to disease severity is based on the judgment of the physician, but even if the patient is asymptomatic or has only mild symptoms, a recommendation for hospitalization and isolation measures may still be given to patients to prevent further spread of the disease in the community ^(7), (8)^.

**Figure 1.**
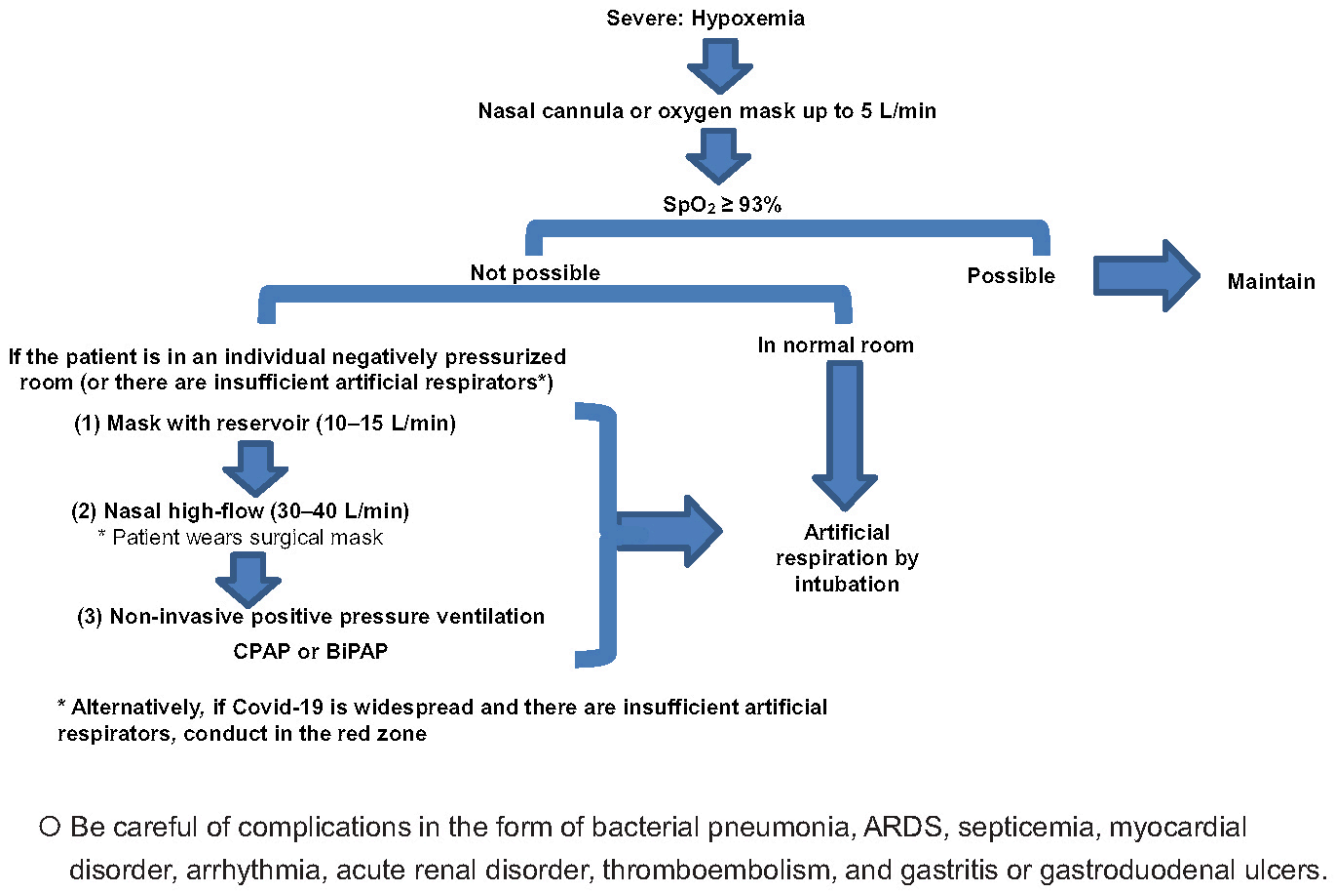
A clinical flowchart to select respiratory support modalities for COVID-19 cases. Ordinarily, SpO_2_ ≥ 93% should be maintained by a nasal cannula up to O_2_ 5 L/min or an oxygen mask up to O2 10 L/min. ^*^Note: When a nasal cannula is used, the patient should wear a surgical mask to suppress aerosol generation. Consider intubation if SpO2 ≥ 93% cannot be maintained even with O2 administration by means of an oxygen mask. In addition, intubation or artificial respiration management should ideally be performed earlier than usual. ^*^Note: Masks with a reservoir (10-15 L/min), nasal high-flow, and non-invasive positive pressure ventilation are normally considered at this stage, but there is a risk of nosocomial infection due to aerosol generation so the use of individual negatively pressurized rooms is desirable. Confirm that the flow is between 30 and 40 L/min when using high-flow and that the cannula is within the nasal cavity. In addition, have the patient wear a surgical mask to suppress aerosol generation.

Respiratory support should be provided by an expert clinician. If a patient suffers from respiratory failure, supplemental oxygen can be provided using a nasal cannula ^(7), (8)^. If SpO2 is still below 93%, high-flow nasal oxygen (HFNO), noninvasive positive pressure ventilation (NPPV), or invasive mechanical ventilation (IMV) is required to maintain appropriate oxygen level. However, HFNO and NPPV potentially produce more contaminated aerosol in the environment, resulting in the nosocomial transmission of SARS-CoV-2 ^(13)^. Thus, a stepwise process of decision-making is helpful to choose the best possible respiratory support modality during an overwhelming situation (Figure 1).

## III．Drug Therapy

Supportive care is still the mainstay of treatment for COVID-19 while there has been a desperate need for drugs to improve prognosis in severe cases. Potentially antiviral, anti-inflammatory, and antithrombotic drugs have been under investigation in clinical trials. In addition, many kinds of medications seem to be utilized on off-label or compassionate use in many countries including Japan ^(8)^.

As of March 2021, no antiviral or potentially active repurposed drugs are recommended for use in patients with mild COVID-19. For patients with moderate to severe disease who require oxygen administration, the combination of remdesivir and steroids (such as dexamethasone) or steroids alone is considered as the standard therapy as well as the best available supportive care ^(7)^.

### 1. Antiviral drugs

Before the COVID-19 epidemic, no anti-coronavirus drugs have been developed; thus, attempts have been made, since the start of the pandemic, to repurpose existing antimicrobial or other potentially effective drugs for this condition. Clinical trials are currently being conducted on drugs that are effective against SARS-CoV-2 in vitro. However, as of March 2021, the number of drugs with established effects remains minimal. In Japan, remdesivir, which received urgent approval from the US Food and Drug Administration in May 2020, has been given special approval. Compassionate use of favipiravir, an anti-influenza drug stockpiled as a countermeasure against a novel strain of influenza virus, is being offered at the physician’s request. Some of the major drugs that are in clinical trials in Japan and overseas are also listed in Table 2 ^(3)^.

**Table 2.** Drug Therapy for COVID-19.

	Developmental status	Drug name	Target patients
Interferon antiviral drug	Special approval	Remdesivir (RNA-dependent RNA polymerase inhibitor)	Moderate to severe cases requiring oxygen administration (less effective in severe cases)
	Off-label use (compassionate use within the framework of observational studies)	Favipiravir (RNA-dependent RNA polymerase inhibitor)	Mild (although efficacy is uncertain)
	Clinical trials	Lopinavir/ritonavir, nelfinavir (HIV protease inhibitor)	No efficacy in critically ill patients
		Hydroxychloroquine (antimalarial drug)	No efficacy
		Interferon	No efficacy
		Ivermectin (antihelminthic drug)	Unclear efficacy
		Ciclesonide (inhaled corticosteroid)	No efficacy
Immune modulator drugs	Approved	Dexamethasone (steroid)	Moderate to severe cases requiring oxygen administration
	Clinical trials	Tocilizumab, sarilumab (genetically modified anti-IL-6 monoclonal antibody)	Unclear efficacy
Immunoglobulin	Clinical trials	Recoveree’s plasma	Unclear efficacy
Antithrombotic drugs	Approved	Heparin	Moderate to severe cases requiring oxygen administration

COVID-19, coronavirus disease; HIV, human immunodeficiency virus

#### i. Remdesivir

This is identified as a nucleic acid analog that was under development as an anti-Ebola virus drug. It acts by inhibiting viral RNA-dependent RNA polymerase. In several studies, a growth inhibitory effect on SARS-CoV-2 was shown ^(14), (15), (16), (17)^. In an international joint clinical trial (double blind) led by the National Institutes of Health in the USA in patients with COVID-19 (moderate to severe), clinical symptoms improved faster in the group administered remdesivir than in the placebo group (10 days vs. 15 days). However, in an international joint clinical trial (single blind) led by the WHO, no reduction in case fatality rate was reported (Table 3). Thus, the effect of this drug is considered minimal in those with severe disease, but it is likely effective in patients with moderate disease. The standard dosage is as follows: for adults, 200 mg should be infused on the first day of administration, while 100 mg should be administered daily from day 2 onward. The standard period of treatment is 5 days.

**Table 3.** Remdesivir Clinical Trials.

Trials	NCT04257656	ACTT-1	GS-US-540-5774	SOLIDARITY
Implementing countries (number of facilities)	China (10)	United States (45), Denmark (8), United Kingdom (5), Greece(4), Germany (3), South Korea (2), Mexico (2), Spain (2), Japan (1), Singapore (1)	United States (45), Italy (11), Spain (9), United Kingdom (8), Germany (6), France (3), France (3), Switzerland (3), Singapore (3), Taiwan (3), Hong Kong (2), Netherlands (1)	World (unknown)
Implementation period	February-March 2020	February-April 2020	March-April 2020	March-October 2020
Study design	Double-blind	Double-blind	Unblind	Unblind
Number of cases	Remdesivir 158 Placebo 79	Remdesivir 541 Placebo 521	Remdesivir (5 days) 197 Remdesivir (10 days) 199 Standard treatment 200	Remdesivir 2743 Standard treatment 2708
Subjects	Requires hospitalization Pneumonia patients	Requires hospitalization Pneumonia patients	Requires hospitalization Patients with pneumonia (SpO_2_ > 94%)	Requires hospitalization Patients
Results	No significant difference in clinical improvement	Faster clinical improvement (10 days vs. 15 days)	On the 11th day of administration, the 5-day treatment group improved symptoms faster than the standard treatment group	No significant difference in mortality

SpO_2_, percutaneous oxygen saturation

In Japan, remdesivir can be used in patients with moderate to severe pneumonia. Since the Ministry of Health, Labour and Welfare of Japan controls the supply of drugs, patient registration must be performed through a dedicated system when this drug is used.

#### ii. Favipiravir

This is an RNA-dependent RNA polymerase inhibitor. This drug has been licensed for pandemic influenza due to a novel strain and stockpiled as a part of pandemic preparedness in Japan. In vitro efficacy has also been shown for SARS-CoV-2, and an investigator-initiated clinical trial on mild cases and a sponsor-initiated clinical trial for NDA on moderate cases (both single blind) have been conducted so far in Japan. In the former, the number of days until a negative PCR test for SARS-CoV-2 was not different from that in the group not administered favipiravir. However, the treated group showed an early tendency of reduced fever though the difference was not statistically siginificant ^(18)^. Another open-label randomized clinical trial conducted in India demonstrated a shorter time to resolution of clinical signs and symptoms although it did not show a shorter time of virus shedding compared with the control group ^(19)^. Compassionate use of favipiravir has been permitted in Japan and other countries. There have been few adverse reactions reported in patients with COVID-19 treated by this drug. However, it is potentially teratogenic and is, thus, contraindicated in pregnant women.

#### iii. Other drugs

The HIV protease inhibitor, lopinavir/ritonavir, has been studied in small clinical trials against severe acute respiratory syndrome (SARS) and MERS (Middle East respiratory syndrome) before the COVID-19 pandemic and showed potential efficacy. However, this drug did not reduce the case fatality recorded in patients with moderate to severe COVID-19 in well-designed clinical trials ^(14), (20)^.

It was revealed that the antimalarial drug hydroxychloroquine is ineffective in reducing mortality and preventing the onset of the disease. Furthermore, well-designed clinical trials have failed to show its efficacy in treatment and post-exposure prophylaxis ^(14), (21)^. Moreover, the WHO has not recommended this drug for COVID-19 in the recent guideline.

### 2. Immune modulator drugs

#### iv. Steroids

Steroids have been used in patients with MERS and SARS, but there have been concerns that they may delay virus clearance and increase case fatality rates. A well-designed clinical trial (RECOVERY) conducted in the UK demonstrated the efficacy of dexamethasone against severe COVID-19 ^(22)^. A significant improvement in prognosis was seen in those requiring invasive mechanical ventilation. The case fatality rate within 28 days of enrolment was 29.0% in the treatment group as against 40.7% in the control group. Also, 21.5% of the dexamethasone-treated group who required oxygen administration at the time of enrolment died within 28 days after enrolment, compared with 25.0% of the control group. However, no prognostic improvement effect was observed in the group that did not require oxygen administration at the time of enrolment. Therefore, this drug is recommended to be administered as the standard therapy for patients with moderate to severe COVID-19 requiring oxygen therapy. The dose is 6 mg of dexamethasone once daily for 10 days. Other steroids probably have the same effect (hydrocortisone 160 mg once daily and methylprednisolone 16 mg every 12 hours) ^(23)^.

#### v. IL-6 inhibitors

As significantly high serum CRP levels are noted in patients with severe COVID-19, the recombinant anti-IL-6 monoclonal antibodies, tocilizumab and sarilumab, have been expected to have immunomodulatory effects and improved prognosis ^(24)^. As of March 2021, at least five clinical trial results have been published as peer-reviewed articles. A clinical trial on critically ill patients in the ICU conducted in the USA showed that tocilizumab (8 mg per kilogram of body weight) and sarilumab (400 mg) produced shorter organ support-free days and improved survival ^(25)^. However, another clinical trial on severe cases conducted in the USA revealed no efficacy ^(26)^.

### 3. Globulin preparation

Clinical trials are being conducted under the hypothesis that the anti-SARS-CoV-2 antibody in the plasma of recovered patients will suppress viral growth and have immunomodulatory effects in patients with the disease. The limited number of studies has been published as of March 2021. One clinical trial conducted in the USA showed that early administration of high-titer convalescent plasma against SARS-CoV-2 to mildly ill infected older adults has reduced the progression of Covid-19 ^(27)^. An observational study which included over 3,000 patients with COVID-19 in the USA supported the survival benefit of high-titer convalescent plasma ^(28)^.

### 4. Antithrombotic drug

Pulmonary thromboembolism can cause respiratory failure and, ultimately, death in patients with COVID-19, with frequent coagulation abnormalities seen in those with moderate and severe diseases requiring oxygen administration ^(29)^. High D-dimer levels are identified as a vital marker of suspected thrombosis. In these patients, anticoagulant therapy, such as heparin, is administered. Low-molecular heparin has not been approved for this indication in Japan.

## Ⅳ. Post-acute Care

Hospitalization recommendations and work restrictions to prevent the spread of the disease will be lifted 10 days after the onset and 3 days after the symptoms improvement ^(7)^. Most recovered patients do not require any further treatment. However, if symptoms are prolonged, such as fatigue, dysosmia, and dysgeusia, the patient should be monitored closely in an outpatient setting ^(30)^. These clinical manifestations called post-COVID syndrome or long COVID are emerging issues and should thus be further examined under a prospective cohort study.

## Ⅴ. Conclusion

It seems that many patients infected with COVID-19 could not receive appropriate medical care because of limited bed availability in hospitals in most countries. Thus, creating a system to observe mildly ill patients, who may suffer from severe disease at later stages, at home or in designated accommodations in the community is a topic that requires consideration. To reduce the case fatality rate, it is vital to ensure standard treatment at an appropriate time. The coordination of healthcare resources in the community is key to reduce overall mortality.

Treatment for COVID-19 is largely supportive, but it is worth noting that many clinical trials have been conducted globally during the pandemic, and some medications have been proven to be effective against COVID-19. There is hope for more effective therapeutics available in the near future.

## Article Information

### Conflicts of Interest

Yasuyuki Kato received consultant fee from Fujifilm Toyama Pharmaceutical, Co.

### Sources of Funding

This work was supported by Health Labour Sciences Research Grant (2020HA20).

### Acknowledgement

I appreciate the board members of JAPAN COVID-19 Case Management Guide Review Committee for their contribution to this work.

### 

The original version is available at https://www.med.or.jp/cme/jjma/newmag/15002/15002.html. The Editors-in-Chief of the Journal of the Japan Medical Association and JMA Journal have permitted the publication of this manuscript.

### 

This is the secondary version of the previous article ^(31)^ published in Japanese on 1 May 2021.
